# Detection of Significant Groups in Hierarchical Clustering by Resampling

**DOI:** 10.3389/fgene.2016.00144

**Published:** 2016-08-08

**Authors:** Paola Sebastiani, Thomas T. Perls

**Affiliations:** ^1^Department of Biostatistics, Boston UniversityBoston, MA, USA; ^2^Geriatrics Section, Department of Medicine, Boston University School of Medicine and Boston Medical CenterBoston, MA, USA

**Keywords:** dendrogram, tree-cutting procedures, resampling techniques

## Abstract

Hierarchical clustering is a simple and reproducible technique to rearrange data of multiple variables and sample units and visualize possible groups in the data. Despite the name, hierarchical clustering does not provide clusters automatically, and “tree-cutting” procedures are often used to identify subgroups in the data by cutting the dendrogram that represents the similarities among groups used in the agglomerative procedure. We introduce a resampling-based technique that can be used to identify cut-points of a dendrogram with a significance level based on a reference distribution for the heights of the branch points. The evaluation on synthetic data shows that the technique is robust in a variety of situations. An example with real biomarker data from the Long Life Family Study shows the usefulness of the method.

## 1. Introduction

Hierarchical clustering is a popular data analysis technique that is commonly used to analyze data sets comprising multiple variables and to identify possible grouping in the data (Murtagh and Contreras, 2012). The associated dendrogram that represents the sorting procedure based on similarity between groups is an effective way to visualize structure in the data and to aid the process of data quality, bias detection, and discovery of informative groups in the data. Hierarchical clustering has become the standard way to display and identify structure in -omics data (Eisen et al., 1998; Hastie et al., 2001; Sebastiani et al., 2003), but despite the name this agglomerative procedure does not provide clusters automatically, and the task of cluster discovery is often based on a subjective decision. “Tree-cutting” procedures can be used to identify subgroups in the data by cutting the dendrogram at some height, and several methods have been proposed to inform this decision based on separation within and between clusters. Examples include the Calinski and Harabasz index (Caliński and Harabasz, 1974), the “Gap statistics” (Tibshirani et al., 2001), and “dynamic tree cutting” (Langfelder et al., 2008). A comprehensive review is presented in Charrad et al. (2014). While these methods provide a solution to the task of selecting the “best set of clusters” among a set of possible choices, they do not provide statistical evidence that there are actually clusters in the data, and whether the set of clusters is statistically significant. In years of experience using hierarchical clustering, the typical question asked by collaborators is to provide the likelihood that the selected clusters are “random.”

Beale (1969) proposed an *F*-statistic to test the hypothesis that a larger set of clusters is significantly better than a smaller one. Model-based clustering provides also a solution by setting cluster membership as a hidden variable, and different model-based clusters can be compared using metrics for model fit such as the Bayesian information criterion (Fraley and Raftery, 2002). This approach has been usually applied to k-means clustering, and it was combined with hierarchical clustering in the context of -omics data (Ramoni et al., 2002b), and Markov chains (Ramoni et al., 2002a). The downside of model-based clustering is that it is parametric, and the solution may not be robust to inappropriate parametric models.

This article proposes a very simple idea to identify “statistically significant” groups in the data using hierarchical clustering. The intuition of the approach is to derive a permutation-based distribution of the similarity between sample profiles under the null hypothesis of no clusters in the data, and then to use quantiles of this reference distribution to cut the dendrogram at heights that would unlikely be seen in random data. The advantages of the proposed approach are that it is easy, it can be used together with existing methods to improve the task of clusters discovery, it is model-free and does not rely on any assumption on the parametric distribution of the data, and it is computationally efficient.

## 2. Methods

Denote by *X* the *n*_*v*_ × *n*_*s*_ data matrix with *n*_*v*_ rows that represent variables and *n*_*s*_ columns that represent sample units. Let the *jth* column of the matrix *X* denote the *profile* of the *jth* sample unit: ${\left(x_{1j},\ldots ,x_{n_{v}j}\right)}^{T}$. We assume that the goal of the analysis is to discover groups of samples that share a similar profile defined by the *v* variables. We focus on hierarchical clustering of the sample units, with Euclidean distance as dissimilarity metric, and complete linkage, so that the shortest distance between two clusters during the agglomerative procedure is defined as the maximum distance between all possible pairs of units in the two clusters.

**Figure 1:**
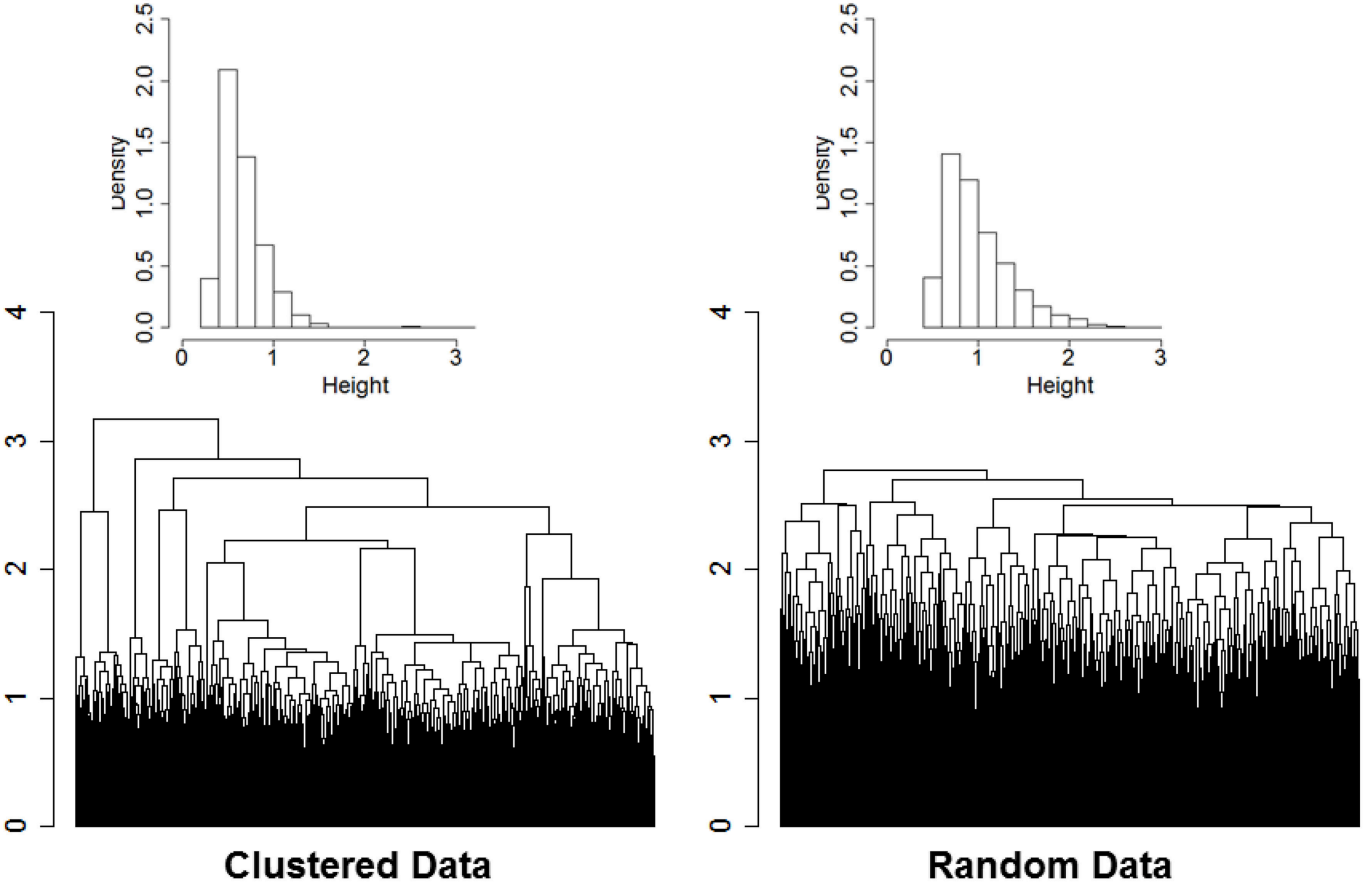
**Dendrogram of hierarchical clustering of 2000 profiles of 16 variables generated from 13 clusters, with cluster size ranging from 2 to 532 (left panel), and dendrogram generated from the same data after reshuffling of the rows (right panel)**. Data were generated from multivariate normal distributions and standardized by row. The histograms describe the distributions of the heights of the branch points.

The rationale of our proposed approach is illustrated by the two dendrograms displayed in Figure 1. The heights of the branch points (clades) in the dendrogram in the left panel are the normalized Euclidean distances used in the agglomerative procedure of *n*_*s*_ = 2000 sample profiles of 16 variables (*n*_*v*_ = 16) simulated from *n*_*c*_ = 13 clusters. Data were generated from multivariate Normal distributions with diagonal variance-covariance matrices, and marginal means that were randomly generated from a Normal distribution with mean 0 and variance 4. Data were standardized by row before using hierarchical clustering, and Euclidean distances were normalized by dividing by the square root of the profile dimension (*n*_*v*_). The dendrogram in the right panel displays the normalized Euclidean distances driving the agglomerative procedure of the data after the elements of each of the 16 rows were reshuffled independently so that there should be no clusters in the data. Even in data with no structure the agglomerative procedure sorts the sample profiles and finds patterns in the data. However, the dendrogram in the left panel is more “dynamic” and the distribution of the distances driving the agglomerative procedure is concentrated on smaller value (median = 0.60) compared to the dendrogram generated from random data (median = 0.91). Furthermore, the distribution of the heights of the branch points in the dendrogram of data with real clusters (inset histogram in the left panel) shows a longer right tail than the distribution of the heights in data with no clusters. The distributions are consistent with the hypothesis that when there are real clusters in the data, profiles in the same cluster should be more similar than profiles from random (unclustered) data, while profiles in different clusters should be more different than profiles from random data.

This example suggests that we could statistically decide if and how many clusters are in a data set by comparing the distribution of the distances used in the agglomerative procedure of hierarchical clustering to a referent distribution generated under the null hypothesis of no clusters in the data. We therefore developed this resampling procedure to identify significant groups using hierarchical clustering:

1. Standardize the data matrix *X* by row to produce the data matrix *Z*, in which the variables are all on the same scale.
2. Conduct hierarchical clustering of the columns of *Z*, and store the *n*_*s*_ − 1 distances from the agglomerative procedure in the vector *D*_*o*_. Normalize the vector of distances by dividing by $\sqrt{n_{v}}$.
3. Repeat *r* times:
  - Reshuffle the elements of each of the *n*_*v*_ rows of the matrix *Z* to produce the data matrix *Z*_*i*_;
  - Conduct hierarchical clustering of the columns of the matrix *Z*_*i*_ and store the vector *D*_*i*_ of *n*_*s*_ − 1 heights from the agglomerative procedure;
4. Compute the reference distributions of heights, say *D*_*e*_, with elements $D_{ej}=\underset{k}{\sum }D_{kj}/r$. Normalize the vector of distances by diving by $\sqrt{n_{v}}$.
5. Display the observed *D*_*o*_ and expected heights *D*_*e*_ in a QQ-plot.
6. Generate significant clusters by cutting the dendrogram displaying the *D*_*o*_ distances at some extreme percentile of the reference distribution. The “significance level” of clusters detected by using the *p*th percentile of *D*_*e*_ is α = 1 − *p*/100.

**Figure 2:**
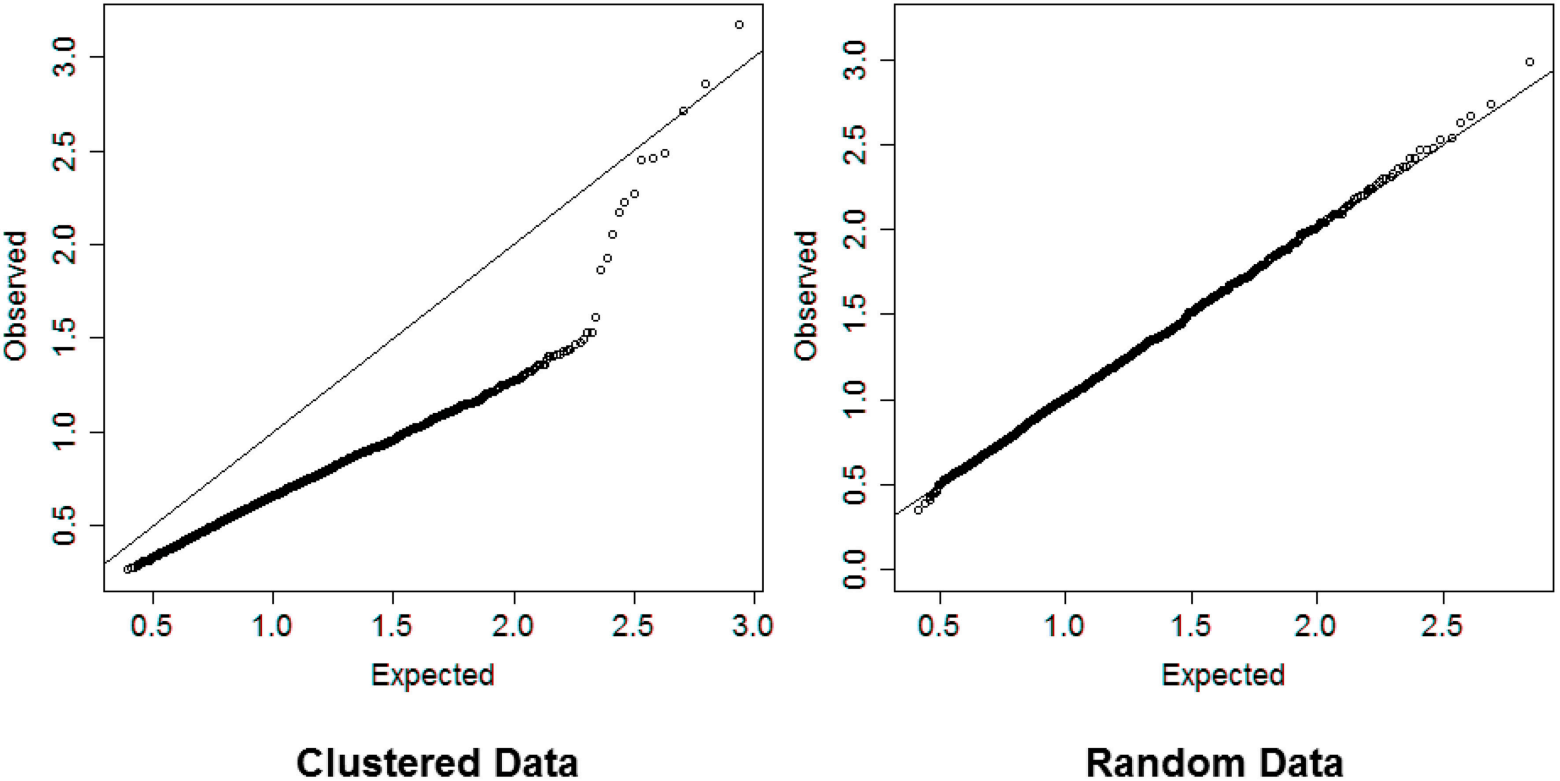
**Left panel:** QQ-plot comparing expected and observed normalized distances used in hierarchical clustering of 2000 profiles of 16 variables generated from 13 clusters. **Right panel:** QQ-plot comparing expected and observed normalized distances used in hierarchical clustering of 2000 profiles of 16 variables generated from only one cluster. The QQ-plot is consistent with the hypothesis that when there are real clusters in the data, profiles in the same cluster should be more similar than profiles from random (unclustered) data and should produce distances that are smaller than what expected in unclusterd data, while profiles in different clusters should be more different than profiles from random data and should produce larger distances than expected by chance. Therefore, a QQ-plot with a clear S-shape as depicted in the plot on the left panel would suggest the presence of very distinct clusters in the data.

In steps 2 and 4, dividing the distances by the square root of the number of variables normalizes the distances so that values are interpretable in general. Inspection of the QQ-plot would inform about the existence of clusters in the data. A situation in which observed and “expected” distances are statistically indistinguishable would suggest that there are no clusters in the data, while departure of the QQ-plot from the diagonal line would suggest that there are clusters, (see Figure 2 for an example). To detect the number of clusters, extreme percentiles of the reference distribution *D*_*e*_ can be used to bound the false detection rate to some fixed value.

## 3. Evaluation

We evaluated the false and true positive rate of the proposed algorithm in data simulated under a variety of scenarios. We also compared the proposed algoritm to the strategy based on Beale F-index (Beale, 1969).

### 3.1. Data generation and analysis

We designed this simulation study to evaluate the impact of the number of true clusters (*n*_*c*_), the number of variables in the data set (*n*_*v*_), the number of profiles in the sample (*n*_*s*_), and the separation of true clusters profiles on the accuracy of the algorithm to detect the correct number of clusters and the correct clusters' composition. For each σ = 2, 5, 10, we generated 10,000 data sets with *n*_*c*_ clusters, *n*_*v*_ variables, and *n*_*s*_ sample profiles. To generate each data set, first the numbers *n*_*c*_, *n*_*v*_ and *n*_*s*_ were randomly selected from ranges 2–20, 2–20, and 1000–5000 respectively. Then, a set of *n*_*c*_ vectors of *n*_*v*_ elements were generated to represent the true profiles of the *n*_*c*_ clusters as follows: One of the true profiles was a vector with all elements equal to 0, while the elements of the remaining *n*_*c*_ − 1 profiles were randomly generated from a Normal distribution with mean 0 and variance σ^2^. Finally, a data matrix *X* was generated with columns that were simulated from multivariate normal distributions with variance-covariance matrix *I*_*n*_*v*__, and mean vector matching one of the *n*_*c*_ true cluster profiles. The number of sample profiles per cluster was also randomly generated. To evaluate the false positive rate of the algorithm, we also generated an additional 10,000 data sets with *n*_*c*_ = 1, while *n*_*v*_ and *n*_*s*_ were randomly selected from ranges 2–20 and 1000–5000 respectively. For each pair (*n*_*v*_, *n*_*s*_), *n*_*s*_ sample profiles based were generated from a multivariate normal distribution with means 0 and variance-covariance matrix $\sigma^{2}I_{n_{v}}$.

In each simulated data set, hierarchical clustering with complete linkage and normalized Euclidean distance was conducted to generated the observed distances *D*_*o*_ used for the agglomerative procedure. Resampling of the rows was conducted 10 times for each data set to derive the reference distribution *D*_*e*_, and percentiles of *D*_*e*_ corresponding to probabilities 0.95, 0.975, 0.99, 0.995, and 0.999 were used to detect clusters. We also used Beale's *F*-statistic to test the global null hypothesis that a given set of clusters are identical vs. the alternative hypothesis that they are not. We iteratively built clusters by cutting the dendrogram at the clades, and we computed the *F*-statistic comparing each new (larger) set of clusters to a single cluster merging all data. If the maximum *F*-statistic was significant (*p*-value < 0.05 or 0.001), we selected the corresponding number of clusters as solution. If no significant result was found among 500 iterations, we selected one cluster as solution.

### 3.2. Metrics

We used a variety of metrics to evaluate the algorithm. A simple calculation of the number of wrong clusters is insufficient because this number would not take into account the range of errors that depends on the number of sample profiles and would also ignore the composition of clusters. We therefore measured the proportion of wrong clusters detected by the algorithm using the ratio:

PWC=(ncq^-nc)/(ns-nc),

where ncq^ is the number of clusters inferred in the data by cutting the dendrogram at the height corresponding to percentile with probabilities, *q* = 0.95, 0.975, 0.99, 0.995, 0.999. The rationale of this metric is that in a sample of *n*_*s*_ profiles, there are at most *n*_*s*_ clusters that can be detected, *n*_*s*_ − *n*_*c*_ of which are wrong, and *PWC* returns the proportion of the possible errors. This metric takes value 0 whenever the algorithm infers the correct number of clusters (ncq^=nc), and takes value 1 whenever the algorithm assigns each sample profile to its own cluster (ncq^=ns). Negative values denote underestimation of the number of clusters, with minimum value (1 − *n*_*c*_)/(*n*_*s*_ − *n*_*c*_) that corresponds to merging all sample profiles into one cluster. When *n*_*c*_ = 1, the *PWC* becomes (ncq^-1)/(ns-1). The metric was modified as (ncq^-nc)/(500-nc) to assess the proportion of wrong clusters detected with the heuristics based on Beale's *F*-statistic.

It is importance to notice that detection of the exact number of clusters does not imply that the algorithm assigns profiles to the right groups. The Rand index proposed in Rand (1971) to measure agreement between two sets of clusters is sensitive to the number of clusters and profiles and can be too optimistic (Solovieff et al., 2010). Therefore, we used two alternative indexes to assess the accuracy of the composition of the clusters inferred by the algorithm: the Cramer's V index (Cramer, 1946), and the average Jaccard's similarity coefficient (Torres et al., 2009). Cramer's V index measures the perfect dependency between the true and inferred clusters' labels and it is calculated as

IC=(χ2/n)/min(nc-1,ncq^-1)

where the χ^2^ statistics is computed in the contingency table cross-classified by the true cluster labels and the cluster labels inferred by the algorithm. The index varies between 0 and 1, with 1 denoting perfect dependency. The limitation of this metric is that when the number of inferred clusters ncq^ differs from the number of true clusters *n*_*c*_, the index can take value 1 as long as there is a perfect dependency in the cross-classification matrix and each row of the contingency table (or each column) has only one element different from 0. Therefore, this index would miss merging of true clusters into larger clusters (See Example 1 in Supplement Material). The Jaccard's index is more appropriate to detect these errors since it is calculated as

$$\begin{array}{l} I_{J}=\left(\underset{ij}{\sum }p_{ij}/q_{ij}\right)/\max\left(r,c\right) \end{array}$$

where *p*_*ij*_ is the number of objects common between true cluster *i* and inferred cluster *j*, and *q*_*ij*_ is the number of objects in either clusters *i* or in cluster *j*. Jaccard's similarity index takes value 1 only when there is perfect dependency between true and inferred clusters and they also match in numbers. However, when the number of inferred clusters differ from the number of true clusters, and some objects are misclassified, the Jaccard's index will be less than one. The two indexes together can inform about the precision to detect clusters, as well as the type of errors. For example, a Cramer's V index equal to 1 and a Jaccard's index less than 1 will suggest that clusters are correctly detected during the agglomerative procedure but some are merged into bigger clusters if the detection rule is too stringent.

### 3.3. Results

#### 3.3.1. False positive rate

**Figure 3:**
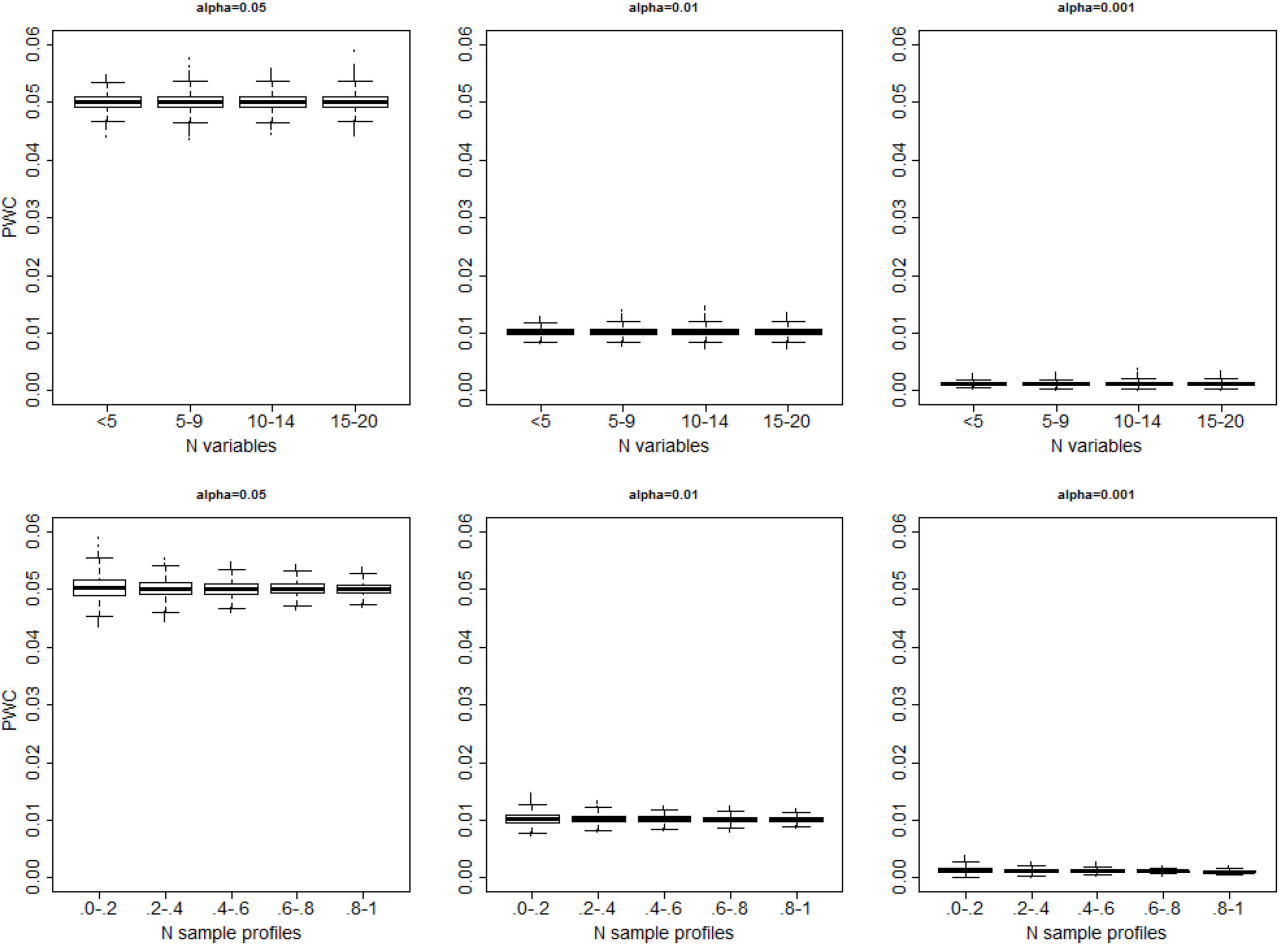
**Distribution of the proportion of falsely detected clusters PWC=(ncq^-1)/(ns-1) for different significance levels (α = 0.05: left panel; α = 0.01; mid panel; α = 0.001 right panel), vs. the number of variables (top panels) and number of sample profiles generated in 10,000 simulations (bottom panels)**. The number of sample profiles are presented in quantiles ranges. In each set, data were generated from one cluster, and cluster detection was based on cutting the dendrogram with observed distances *D*_*o*_ using the percentiles of the resampling-based reference distribution *D*_*e*_.

In all 10,000 simulated data sets with only one cluster the algorithm always generated some partition of the sample profiles. The distributions of the proportion of wrong clusters for significance levels 0.05, 0.01 and 0.001, and σ^2^ = 4 are displayed in Figure 3. Supplement Figure 1 displays a more comprehensive set of results. The proportion of errors shown by *PWC* matches the expected error rates for different choices of the quantiles used to cut the dendrogram, and is independent of the number of variables in the simulated data set. Beale *F*-test correctly assigned the sample profiles to one cluster in 53% of the simulated data sets when the level of significance was 0.05, and in 59% of simulated data sets when the level of significance was 0.001. However, in the remaining cases, the *F*-statistic kept increasing with larger number of clusters and produced a *PCW* = 1. The proportion of wrong clusters appeared to increase with the number of variables (See Supplement Figure 2).

#### 3.3.2. True positive rate

**Figure 4:**
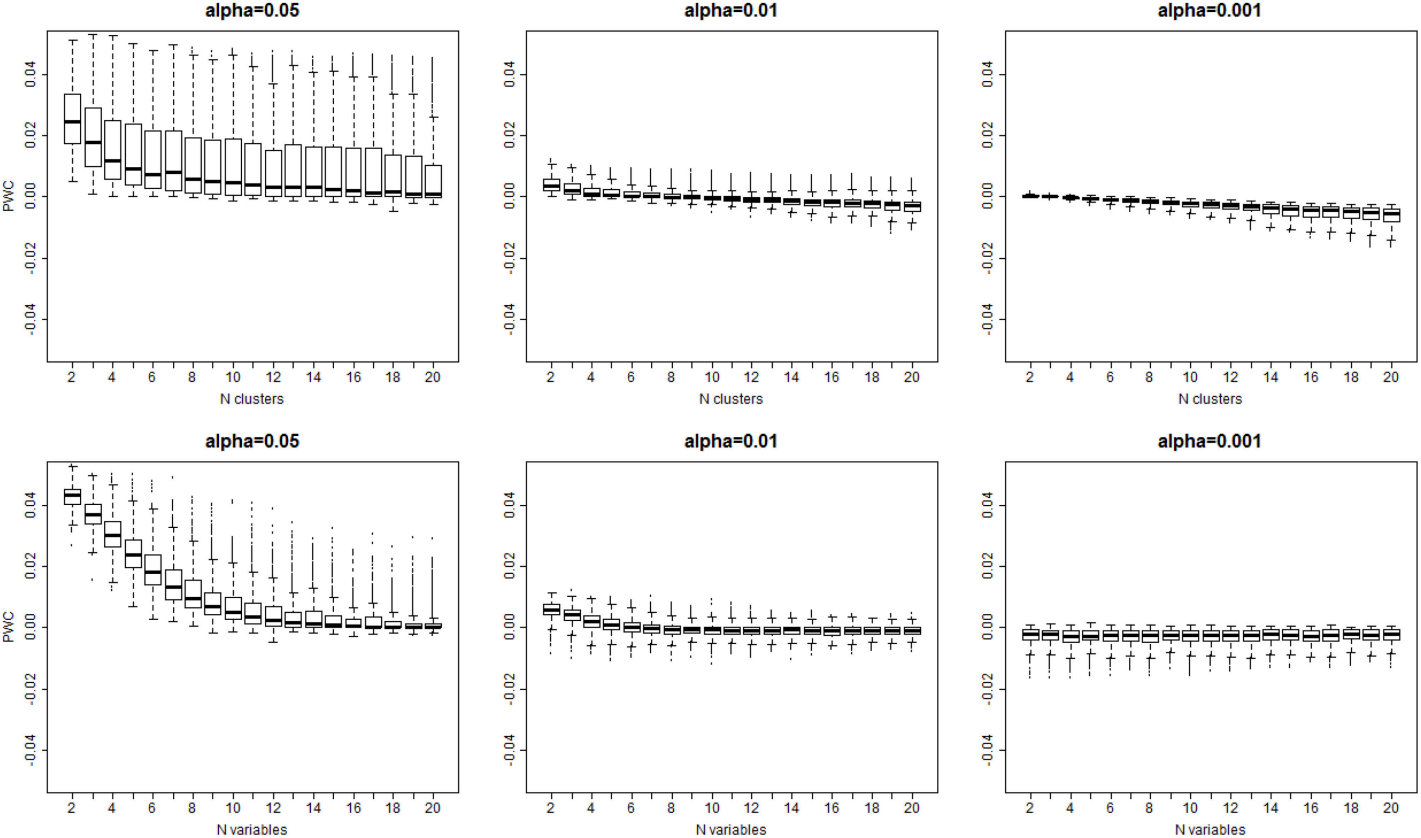
**Distribution of the error rate (PCW=(ncq^-nc)/(ns-nc)) vs. the true number of clusters (top panel), and the true number of variables bottom panel) for different significance levels α = 0.05, 0.01, 0.001**. The error rate decreases with larger number of variables and larger number of clusters that are both associated with larger separation of clusters (See Supplement Figure 3).

**Figure 5:**
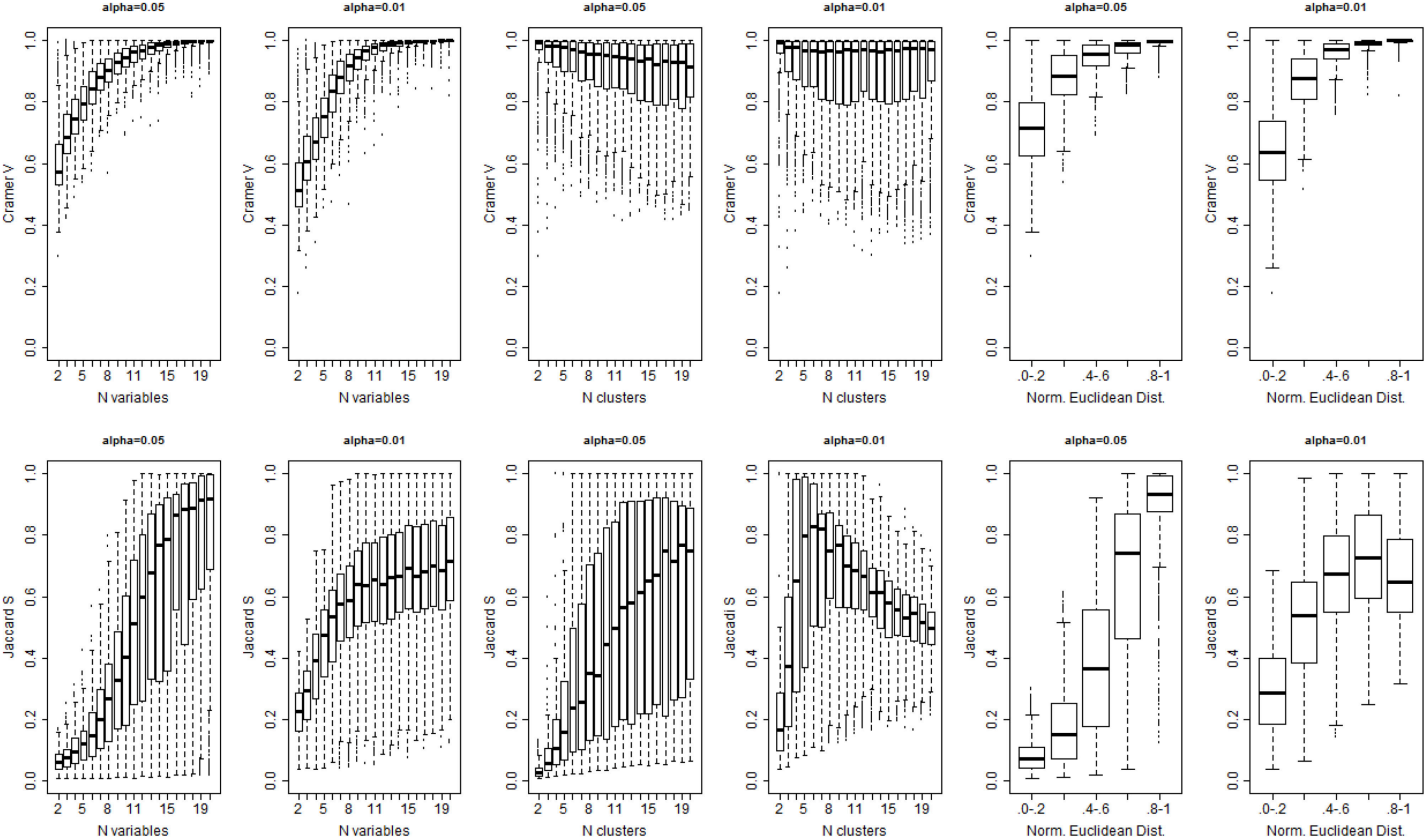
**Cramer's Index V (top panel) and Jaccard's Similarity Index (bottom panel) for different levels of significance α, numbers of variables (columns 1 and 2), number of true clusters (columns 3 and 4), and separation between true profiles (Norm. Euclidean Dist.)**.

The algorithm detected the correct number of clusters only in 11% of simulated data sets when a 0.05 significance level was used, in only 9% of cases with 0.01 significance level, and in only 8% of cases with 0.001 significance level. Figure 4 shows the *PWC* vs. the number of true clusters (*n*_*c*_) and the number of variables (*n*_*v*_), for 5, 1, and 0.1% significance levels and σ = 2. More comprehensive results are shown in Supplement Figure 3. The *PWC* tends to decrease with increasing numbers of true clusters and variables. This result is consistent with the observation that the normalized Euclidean distance between true profiles in the 10,000 simulations increases with the number of variables (Supplement Figure 4) so that the clusters become easier to detect and, consistently, the precision of the algorithm increases. The use of more extreme percentiles may underestimate the correct number of clusters and therefore less extreme percentiles should be used for cluster detection when the distance between *D*_*o*_ and *D*_*e*_ is large. This observation is also emphasized by the results in Figure 5, and also Supplement Figures 5–7, that show increasing Cramer's V Index and Jaccard's similarity index for increasing number of variables that define the sample profiles (columns 1 and 2), increasing number of clusters (columns 3 and 4) and increasing separation between true profiles used to generate the data (column 5 and 6). However, Jaccard's similarity between true cluster and inferred cluster labels tend to decrease if the dendrogram is cut at a too extreme height and consequently some clusters are merged into larger ones. Beale F ratio detected the correct number of clusters in approximately 26% of cases, but the *PWC* in the remaining 74% of simulated data was high and in approximately 25% of simulated dataset the algorithm inferred 500 clusters (Supplement Figure 8).

### 3.4. Discussion of the simulations results

The simulation study suggests that the algorithm provides a reasonable heuristics to detect significant groups from hierarchical clustering although the accuracy of the results can vary with the choice of percentile value used to cut the dendrogram. Compared to Beale's *F*-ratio, using the empirical distribution of the dendrogram heights appears to be more reliable, particularly when the number of variables or the number of clusters in the data are large. The choice of the best detection threshold can be informed by the magnitude of the difference between the observed and expected distances *D*_*o*_ and *D*_*e*_ displayed in a QQ-plot. Substantial separation between *D*_*o*_ and *D*_*e*_ would indicate that a moderate detection threshold, for example the 95th percentile, should be sufficient to detect the correct number of clusters and assign correct cluster membership to the sample profiles. For example, the QQ-plot in the left panel of Figure 2 show substantial separation between the set of distances *D*_*o*_ and *D*_*e*_ for the data used in the dendrogram in Figure 1. Cutting the dendrogram at a height = 1.76 (95th percentile of *D*_*e*_) detects exactly 13 clusters, with only 1 of the 2000 profiles that was wrongly classified, and a similarity metric *S*_*J*_ = 0.97. Cutting the dendrogram at more extreme heights reduces the true discovery rate, for example only 9 clusters are detected using the 99th percentile of the referent distribution. More limited separation between real clusters makes both tasks of detecting the correct number of clusters and assigning the correct cluster membership more challenging. In these situations, we advocate using more extreme percentiles from the distribution of *D*_*e*_ for cluster detection. The next application to real data illustrates this point.

## 4. Application

**Figure 6:**
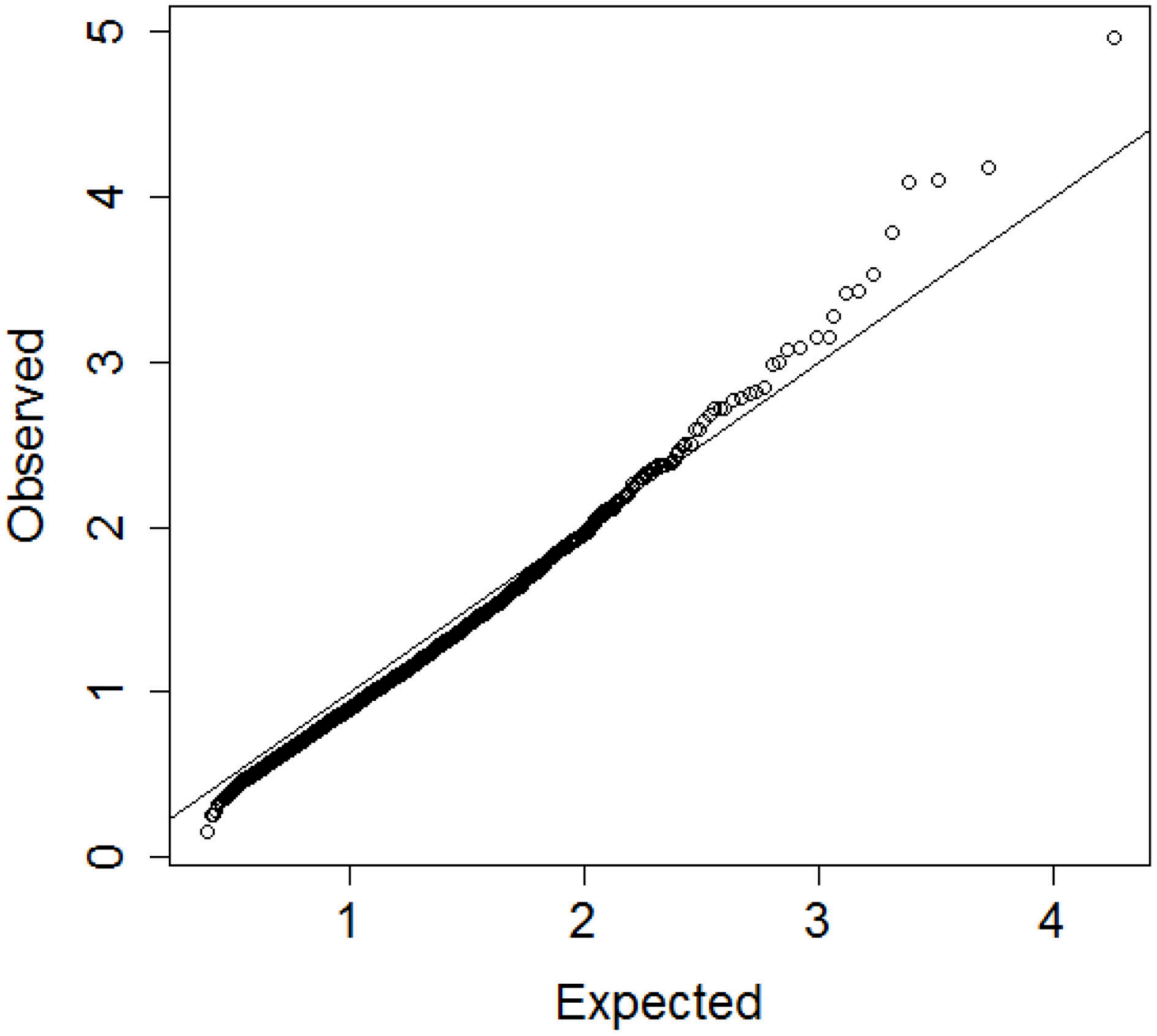
**QQplot of the observed heights of the branch nodes in the dendrogram of hierarchical clustering of 4704 profiles of 19 biomarkers in participants of the Long Life Family Study (y-axis) and the expected heights based on 10 resampling of the data**. The departure from the diagonal line suggests that there are significant clusters in the data.

Prior studies by us and others indicate that circulating biomarker values correlate with physical function, anabolic response and healthy aging (Stenholm et al., 2010; Banerjee et al., 2011; Newman et al., 2011). While analysis of individual biomarker levels is often confounded by diverse underlying physiological states resulting in poor specificity, analysis of multiple biomarkers simultaneously could discover robust signatures of key circulating factors that distinguish between different patterns of aging, including early frailty and healthy aging. Consistent with this hypothesis, we identified 19 blood biomarkers that include some tests from total blood counts, lipids, markers of inflammation and frailty measured in 4704 participants of the Long Life Family Study (LLFS; Newman et al., 2011), and used the proposed algorithm to group LLFS participants into clusters characterized by different patterns of biomarkers. We used hierarchical clustering of age and sex standardized biomarkers and generated the vector of 4703 distances *D*_*o*_. Resampling of the biomarker data was conducted 10 times to generate the reference set of distances *D*_*e*_. Figure 6 shows the QQ-plot of *D*_*o*_ and *D*_*e*_ and the departure from the diagonal line suggests that there are indeed significant clusters in the data, although the separation between *D*_*o*_ and *D*_*e*_ is limited and extreme percentiles should be used for guaranteeing accuracy. We therefore investigated clusters that are detected for extreme percentiles corresponding to probabilities *p* = 0.99, 0.991, …, 0.999 of the reference distribution *D*_*e*_.

**Figure 7:**
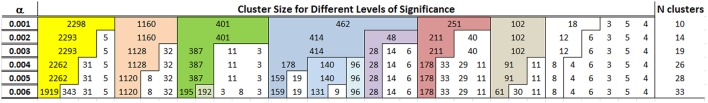
**Clusters detected by cutting the dendrogram using different percentiles of the the reference distribution ***D***_***e***_ in the LLFS data**. The first column shows the significance level α = 1 − *p* where *p* was used to determine the percentiles of the reference distribution *D*_*e*_. The other columns report the size of different clusters and colors track clusters that are robust with respect to different percentiles. For example, the algorithm detects 10 clusters for α = 0.001, and the largest cluster in yellow includes 2298 profiles. The bulk of this cluster is maintained when the algorithm detects 14 clusters with α = 0.002, and the 2298 profiles are split into a cluster with 2293 profiles and a smaller cluster with only 5 profiles.

Figure 7 shows the composition of clusters detected for *p* = 0.994, 0.995, …, 0.999. As the probability of Type I error increases, so does the number of clusters. However, the number of additional clusters introduced for more liberal thresholds is small and most of the differences are in the generation of new clusters with very small number of individuals. With a Type I error rate of 0.4% the algorithm detects 26 clusters, and the most noticeable difference from the clusters detected with a Type I error rate ranging between 0.1 and 0.3% is the split of the cluster with 462 participants into 3 smaller clusters of 96, 140 and 178 participants. These clusters are highlighted in blue in Figure 7. For all subsequent analyses, we used the 26 clusters detected for a significance level of 0.4% that provides a good compromise between number of clusters and expected error rate.

Each cluster was characterized by the associated profile (or signature) of the 19 biomarkers that is defined by the vector of means and standard deviation of the biomarker data in the subjects allocated to the specific cluster. The most common profile corresponding to the largest cluster was shared by about 50% of LLFS participants (highlighted in yellow in Figure 7) and was characterized by biomarkers that, on average, match the values expected for the age and sex of cluster members. We selected this profile as the *referent profile* and we described it as representing “average aging” in LLFS. Another profile shared by about 25% of LLFS participants is characterized by a subset of the 19 biomarkers that tend to be below the values observed in the referent profile. Other profiles shared by smaller proportions of LLFS participants are characterized by varying combinations of subsets of biomarkers that tend to be above or below the normal aging values. We analyzed the predictive values of these biomarker profiles by associating them with survival rates and risk of age related diseases including cancer, cardiovascular events, and type 2 diabetes using about 7 years of follow up data in LLFS. All analyses were conducted using Cox proportional hazard model, were stratified by sex, and were adjusted by the age of participants at enrollment in the study. The analysis showed that one biomarker profile was associated with a statistically significant reduction in hazard for mortality and type 2 diabetes relative to the referent profile, while other 9 profiles were associated with less successful aging, characterized by higher risk for morbidity and mortality. Interestingly, the clusters of 140 and 178 subjects that are generated by choosing the percentile 99.6% are associated with a similar increased risk for diabetes compared to the referent profile, but different risk for mortality. The predictive values of 7 of these 10 profiles was replicated in an independent data set from the Framingham Heart Study with statistically significant and consistent effects, while the other 3 profiles showed consistent effects but did not reach statistical significance associations. The complete analysis will be described elsewhere.

## 5. Discussion

We proposed a very simple method to identify statistically significant groups in data comprising multiple variables using hierarchical clustering. The method consists of generating a permutation-based distribution of the similarity between sample profiles under the null hypothesis of no clusters in the data, and then uses quantiles of this reference distribution to cut the dendrogram at an height that would unlikely be seen in random data. A large simulation study showed that the algorithm provides a reasonable heuristics to detect significant groups from hierarchical clustering although the accuracy of the results can vary with the choice of percentile value used to cut the dendrogram. We also showed that a QQ-plot of the observed and expected heights of the branch nodes in the dendrogram can inform about the presence of clusters in the data and the magnitude of percentiles that would likely produce accurate results. We applied the algorithm to detect clusters defined by patterns of 19 blood biomarkers in a sample of 4704 participants of the LLFS. The analysis identified several profiles of biomarkers that are associated with varying types of aging in LLFS participants. The replication of some of these association in an independent data set from the Framingham Heart Study suggests that the algorithm works well in practice.

Hierarchical clustering is a very popular method that is easy to visualize and to describe to a non-statistical audience. We believe that our proposed algorithm provides a statistically sound method for the discovery of significant clusters that maintains the simplicity of the overall approach. In addition the algorithm is easy to implement (See an example of R script in the Supplement material) and it is computationally efficient. In practice we noted that resampling the data 10 times is sufficient to generate a reliable reference distribution while maintaining computational efficiency when the number of sample profiles is large. In our evaluation we tried resampling up to 1000 times but we did not see noticeable differences in the results. The accuracy to estimate the reference distribution depends also on the number of sample profiles, and with a small number of profiles there will be a limit to the level of significance that can be determined with the resampling procedure. Although we focused attention to hierarchical clustering with complete linkage and Euclidean distance as dissimilarity metric, the approach that we have proposed can be applied to hierarchical clustering with different dissimilarity and linkage choices.

An important preliminary step of the analysis is the standardization of the rows of the data matrix so that all the variables are on the same scale. Since hierarchical clustering with complete linkage is sensitive to outliers, it is advisable to remove outliers before the analysis, using for example principal component analysis (Jolliffe, 2002). We have also noticed that standardization of the variable profiles using trimmed means may lead to more robust results.

Theoretically it should be possible to derive a closed-form solution for the reference distribution *D*_*e*_ assuming that the data follow special probability distributions, such as a Normal distribution. However, an important feature of the proposed method is that it is model-free and in many applications with biological data, standard probability distributions may fail to capture the complexity of the data. Furthermore, the QQ-plot of observed and expected distances also provide a simple but effective way to decide whether there are significant clusters in the data.

Our method differs substantially from the approach of “bootstrap *p*-values” implemented in the R package *pvclust* (Suzuki and Shimodaira, 2006), that provides a level of significance for each clade of the dendrogram. Similar approaches to detect the significance levels of each individual cluster have been proposed in (Levenstien et al., 2003; Park et al., 2009). Our approach provides a simple heuristic to decide whether there are clusters, and where to cut the dendrogram to detect these clusters. The method we propose does not assess the significance of individual clusters, but tests only the overall significance of a set of clusters or, in other words, the global hypothesis that there are clusters in the data. It would be interesting to combine the two approaches and we conjecture that our algorithm could be used to reduce the computation time of *pvclust* that at the moment can be substantial in large datasets. Similarly, the simulations suggested that Beale's index could be used in conjunction with the proposed approach to reduce the number of falsely discovered clusters.

In addition to the discovery of biomarker profiles of aging in LLFS, we have used the proposed algorithm to detect significant clusters in other -omic data with encouraging results. Following the very successful application of hierarchical clustering to discover subtypes of lymphoma (Alizadeh et al., 2000), this clustering method has become one of the most commonly used techniques to analyze -omics data, and to discover new disease subtypes using gene expression and other gene product data. Although many limitations of hierarchical clustering are well-known (Quackenbush, 2001), the most critical limitation is the lack of statistical rules to detect clusters with some measure of uncertainty, and this limitation is often overlooked. In the simulations and the application to real data, we used the algorithm to cluster data sets with a number of profiles that is comparable to the number of expressed genes in many gene expression data sets (*n*_*s*_ ranged between 1000 and 5000). We also used the algorithm for clustering larger data sets with up to 20,000 profiles with comparable results. The approach proposed here could become a useful and simple way for discovering new biological processes and disease conditions from -omic data with higher specificity.

## Author contributions

PS designed the study, developed the method and drafted the manuscript. TP provided data for evaluation and contributed to manuscript writing.

## Funding

This work was supported by the National Institute on Aging (NIA cooperative agreements U01-AG023755 to TP).

### Conflict of interest statement

The authors declare that the research was conducted in the absence of any commercial or financial relationships that could be construed as a potential conflict of interest.
