# Supplementary material for: Detection of Significant Groups in Hierarchical Clustering by Resampling
Source: Front Genet. 2016 Aug 8;7:144. doi: 10.3389/fgene.2016.00144 (PMC4976109; doi:10.3389/fgene.2016.00144)
Supplement: Supplementary file 1 [file Presentation1.PPTX]

## Slide 1
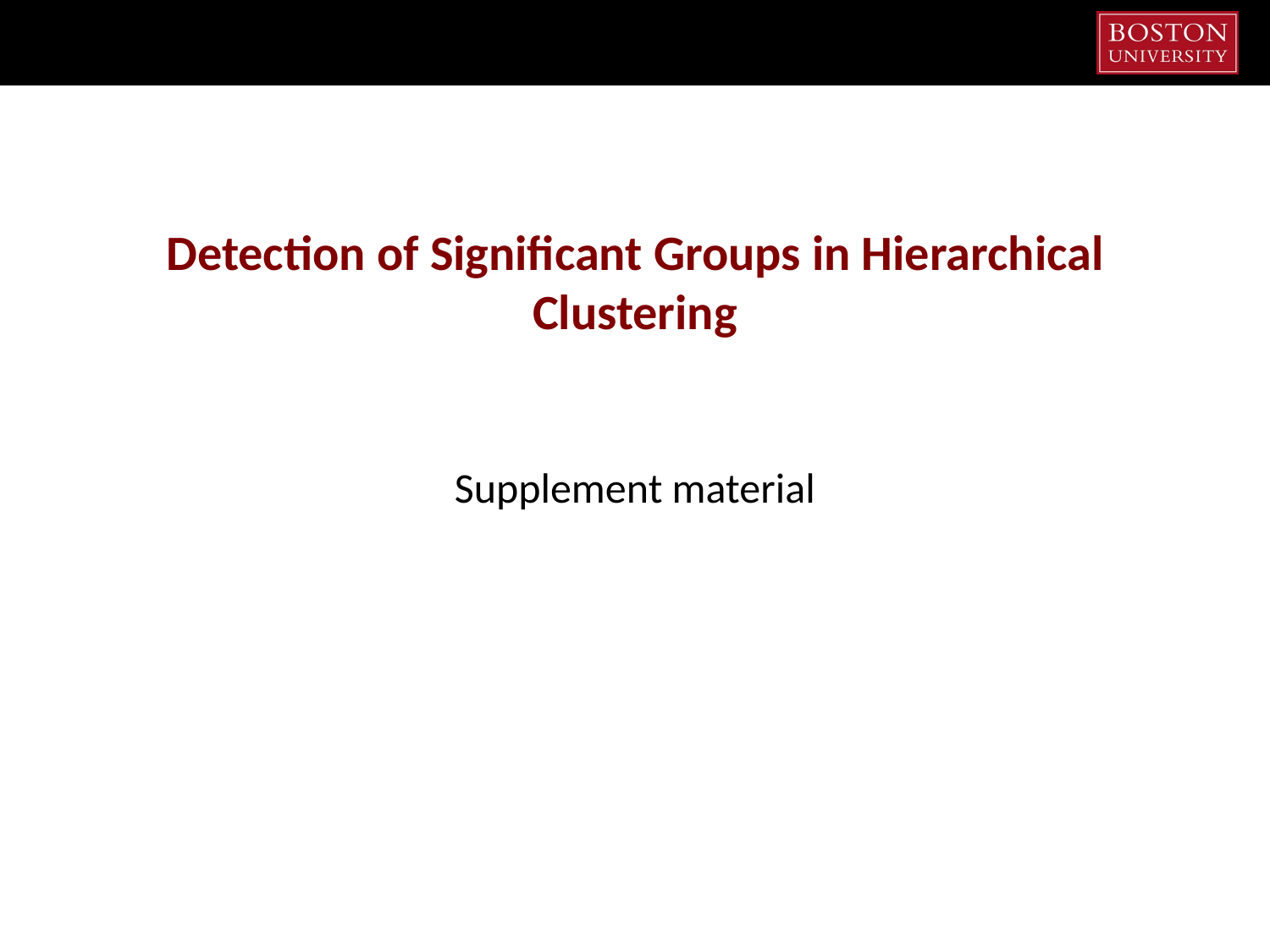

# Detection of Significant Groups in Hierarchical Clustering
Supplement material

## Slide 2
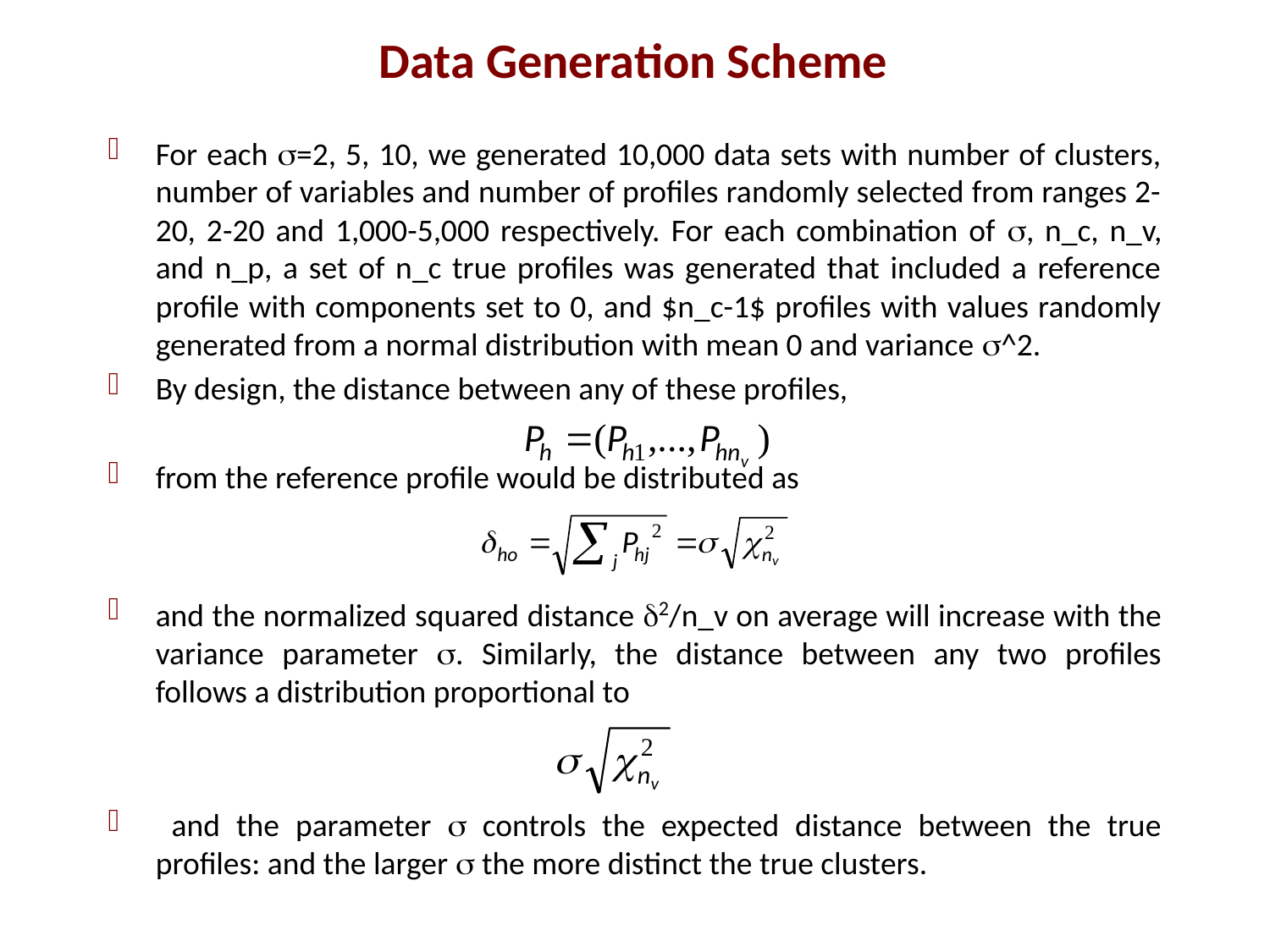

# Data Generation Scheme
For each =2, 5, 10, we generated 10,000 data sets with number of clusters, number of variables and number of profiles randomly selected from ranges 2-20, 2-20 and 1,000-5,000 respectively. For each combination of , n_c, n_v, and n_p, a set of n_c true profiles was generated that included a reference profile with components set to 0, and $n_c-1$ profiles with values randomly generated from a normal distribution with mean 0 and variance σ^2.
By design, the distance between any of these profiles,
from the reference profile would be distributed as
and the normalized squared distance 2/n_v on average will increase with the variance parameter . Similarly, the distance between any two profiles follows a distribution proportional to
 and the parameter  controls the expected distance between the true profiles: and the larger  the more distinct the true clusters.

## Slide 3
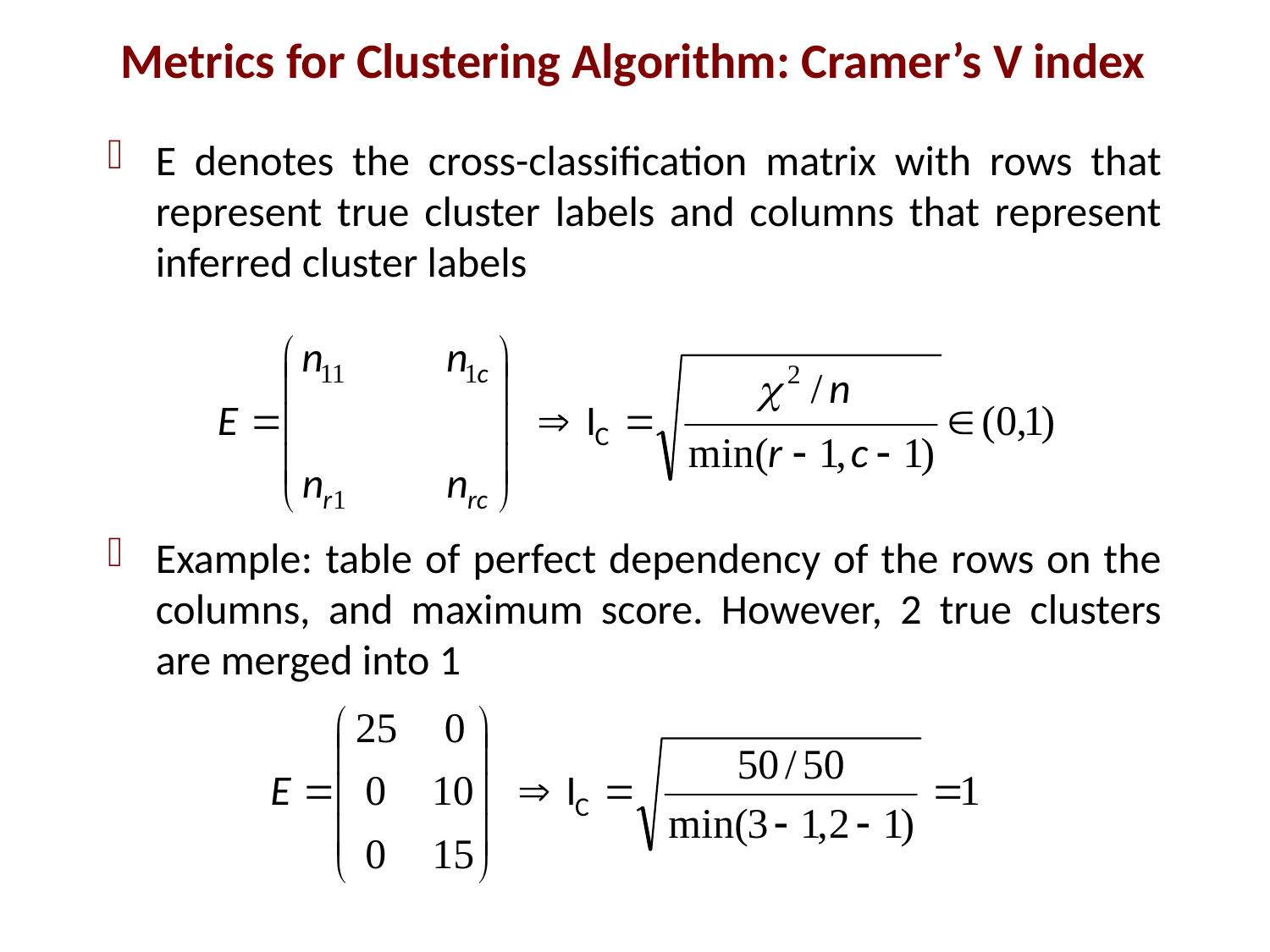

# Metrics for Clustering Algorithm: Cramer’s V index
E denotes the cross-classification matrix with rows that represent true cluster labels and columns that represent inferred cluster labels
Example: table of perfect dependency of the rows on the columns, and maximum score. However, 2 true clusters are merged into 1

## Slide 4
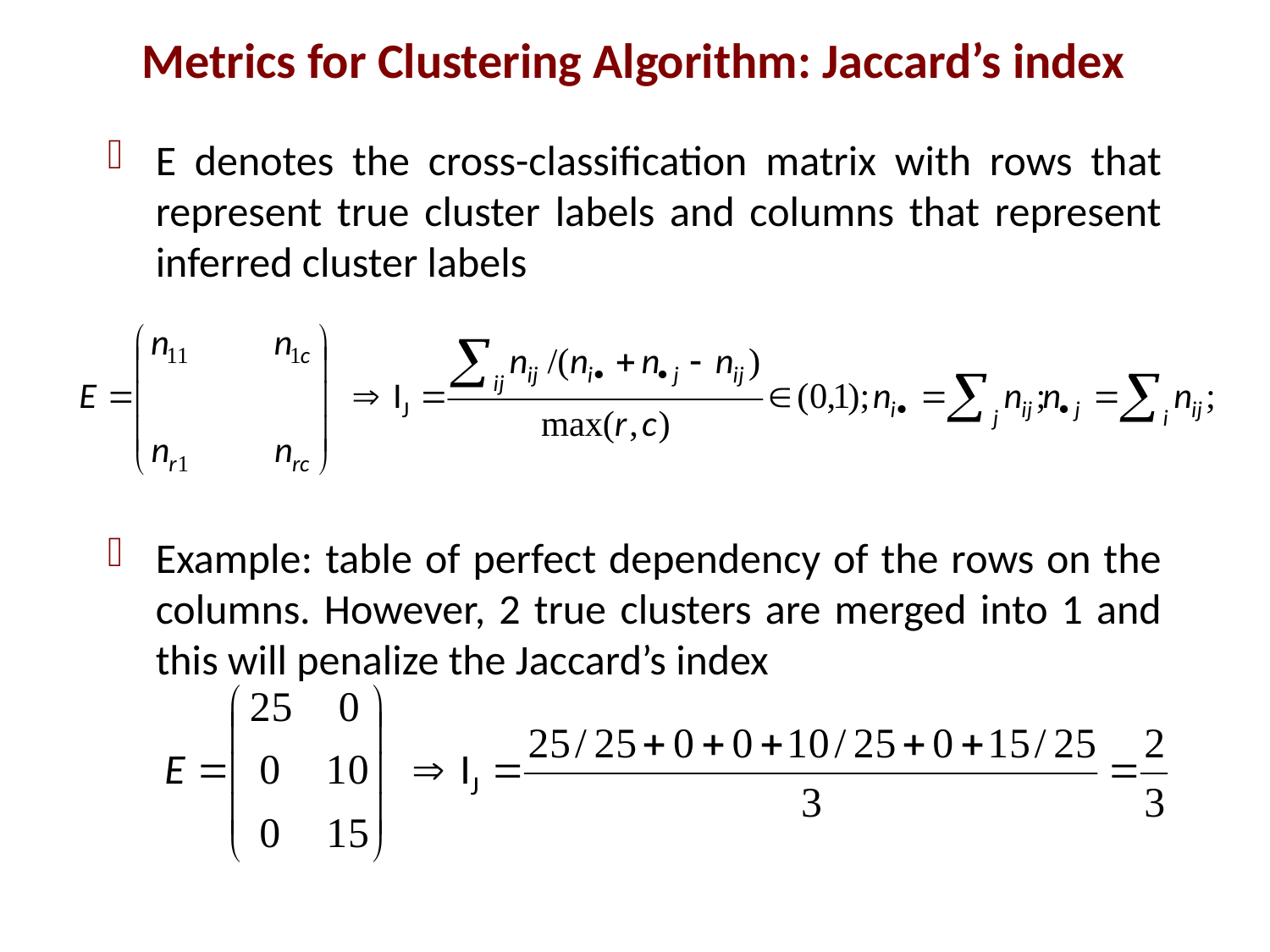

# Metrics for Clustering Algorithm: Jaccard’s index
E denotes the cross-classification matrix with rows that represent true cluster labels and columns that represent inferred cluster labels
Example: table of perfect dependency of the rows on the columns. However, 2 true clusters are merged into 1 and this will penalize the Jaccard’s index

## Slide 5
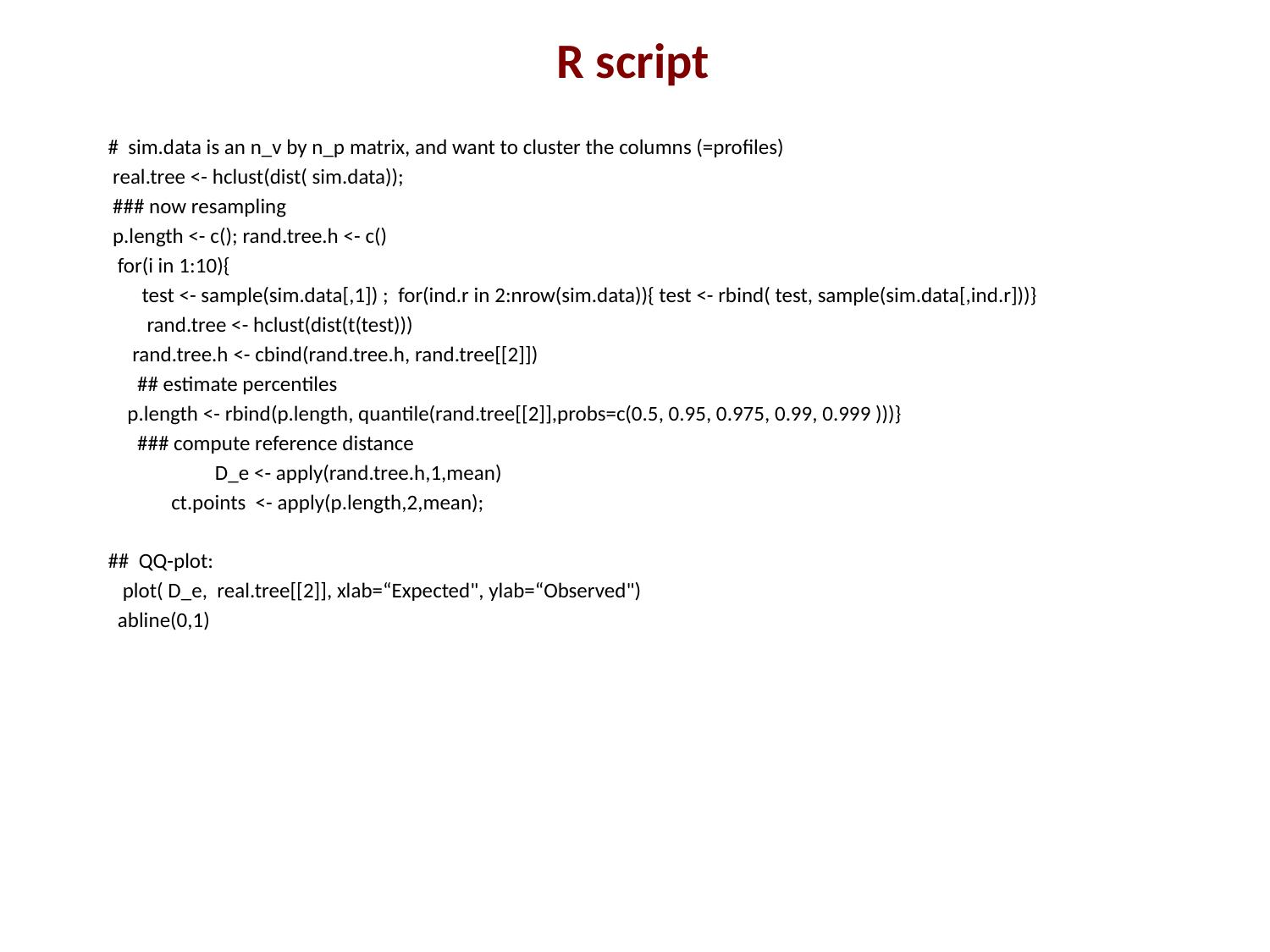

# R script
# sim.data is an n_v by n_p matrix, and want to cluster the columns (=profiles)
 real.tree <- hclust(dist( sim.data));
 ### now resampling
 p.length <- c(); rand.tree.h <- c()
 for(i in 1:10){
 test <- sample(sim.data[,1]) ; for(ind.r in 2:nrow(sim.data)){ test <- rbind( test, sample(sim.data[,ind.r]))}
 rand.tree <- hclust(dist(t(test)))
 rand.tree.h <- cbind(rand.tree.h, rand.tree[[2]])
 ## estimate percentiles
 p.length <- rbind(p.length, quantile(rand.tree[[2]],probs=c(0.5, 0.95, 0.975, 0.99, 0.999 )))}
 ### compute reference distance
 D_e <- apply(rand.tree.h,1,mean)
 ct.points <- apply(p.length,2,mean);
## QQ-plot:
 plot( D_e, real.tree[[2]], xlab=“Expected", ylab=“Observed")
 abline(0,1)

## Slide 6
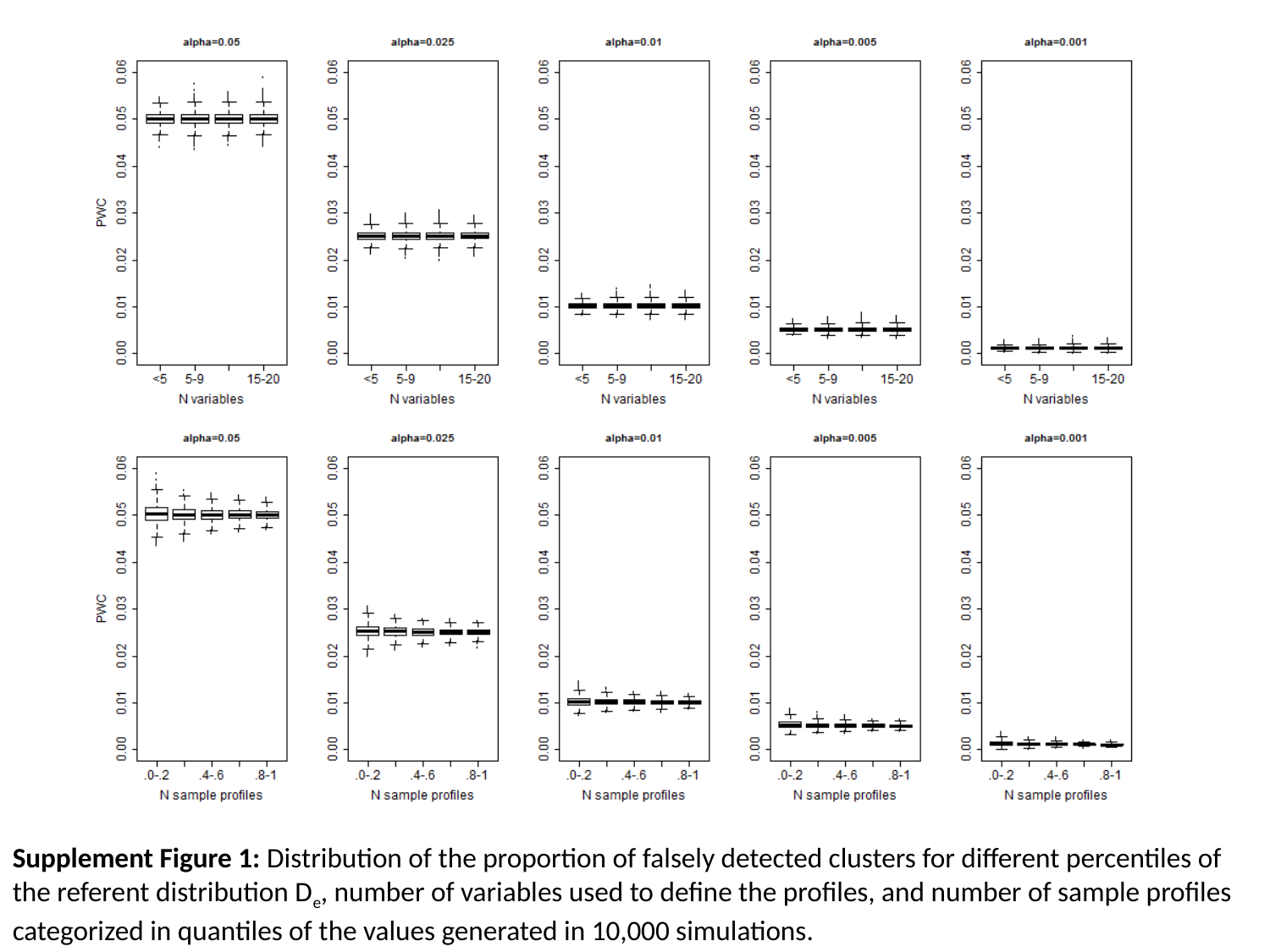

Supplement Figure 1: Distribution of the proportion of falsely detected clusters for different percentiles of the referent distribution De, number of variables used to define the profiles, and number of sample profiles categorized in quantiles of the values generated in 10,000 simulations.

## Slide 7
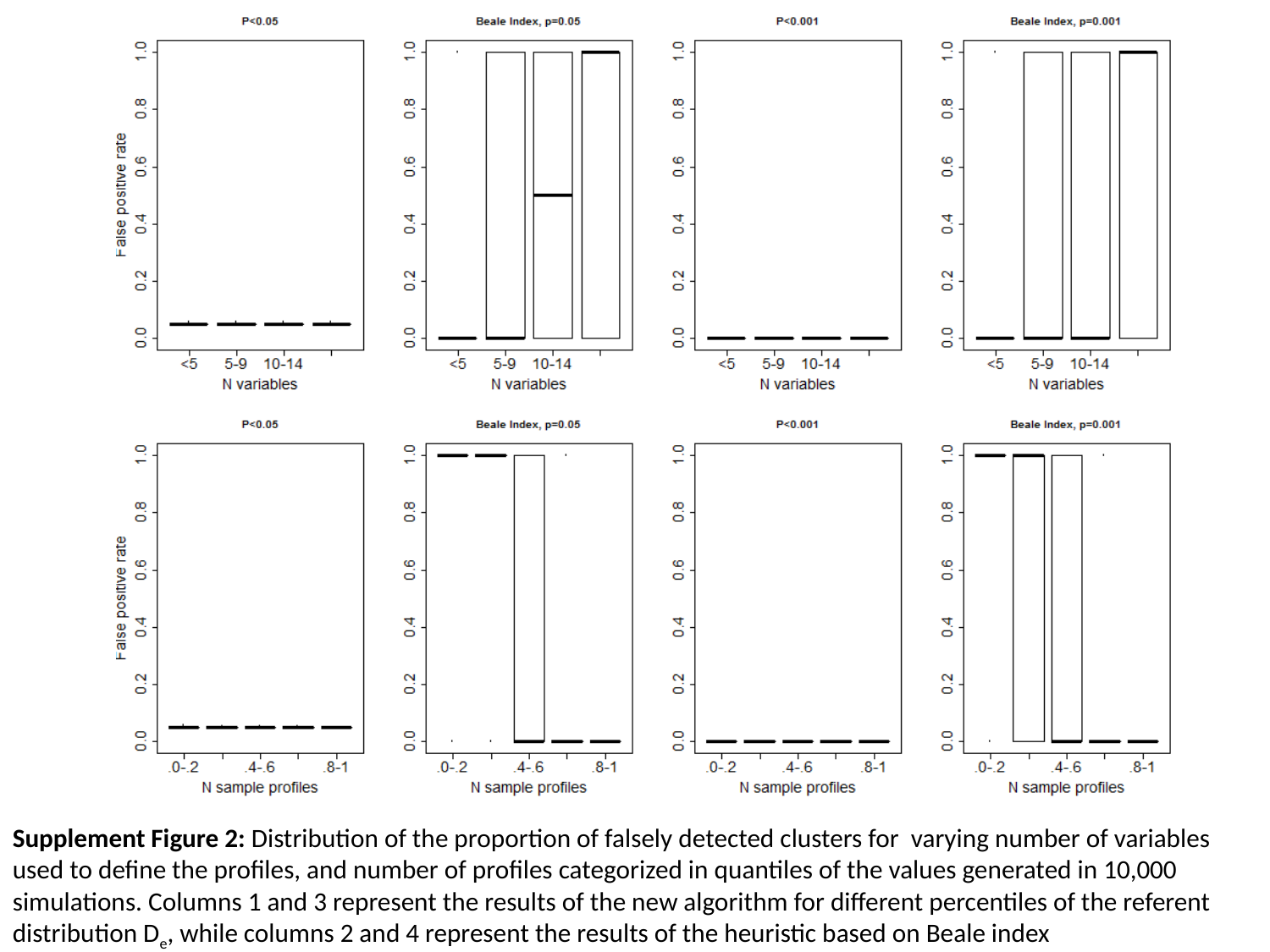

Supplement Figure 2: Distribution of the proportion of falsely detected clusters for varying number of variables used to define the profiles, and number of profiles categorized in quantiles of the values generated in 10,000 simulations. Columns 1 and 3 represent the results of the new algorithm for different percentiles of the referent distribution De, while columns 2 and 4 represent the results of the heuristic based on Beale index

## Slide 8
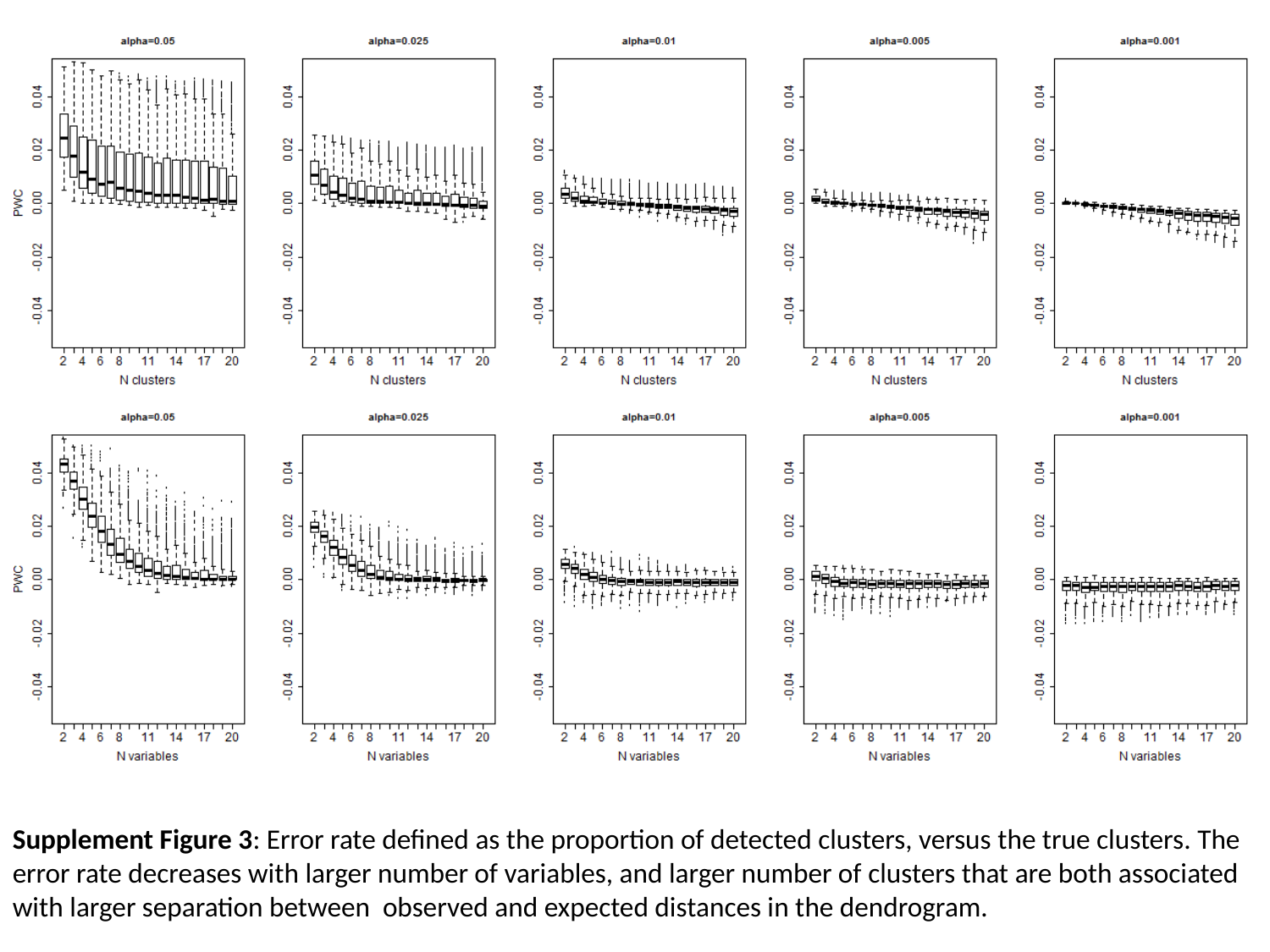

Supplement Figure 3: Error rate defined as the proportion of detected clusters, versus the true clusters. The error rate decreases with larger number of variables, and larger number of clusters that are both associated with larger separation between observed and expected distances in the dendrogram.

## Slide 9
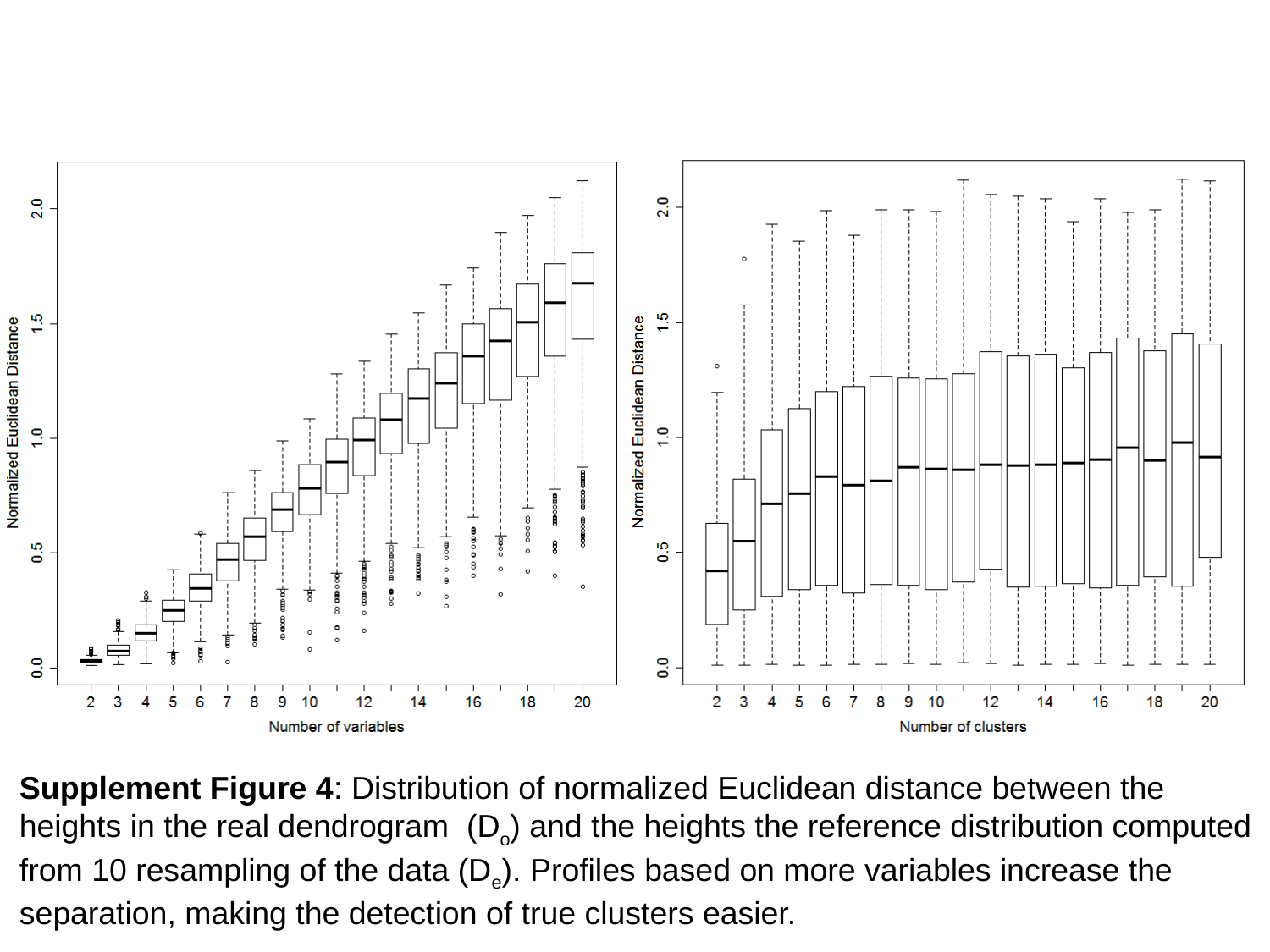

Supplement Figure 4: Distribution of normalized Euclidean distance between the heights in the real dendrogram (Do) and the heights the reference distribution computed from 10 resampling of the data (De). Profiles based on more variables increase the separation, making the detection of true clusters easier.

## Slide 10
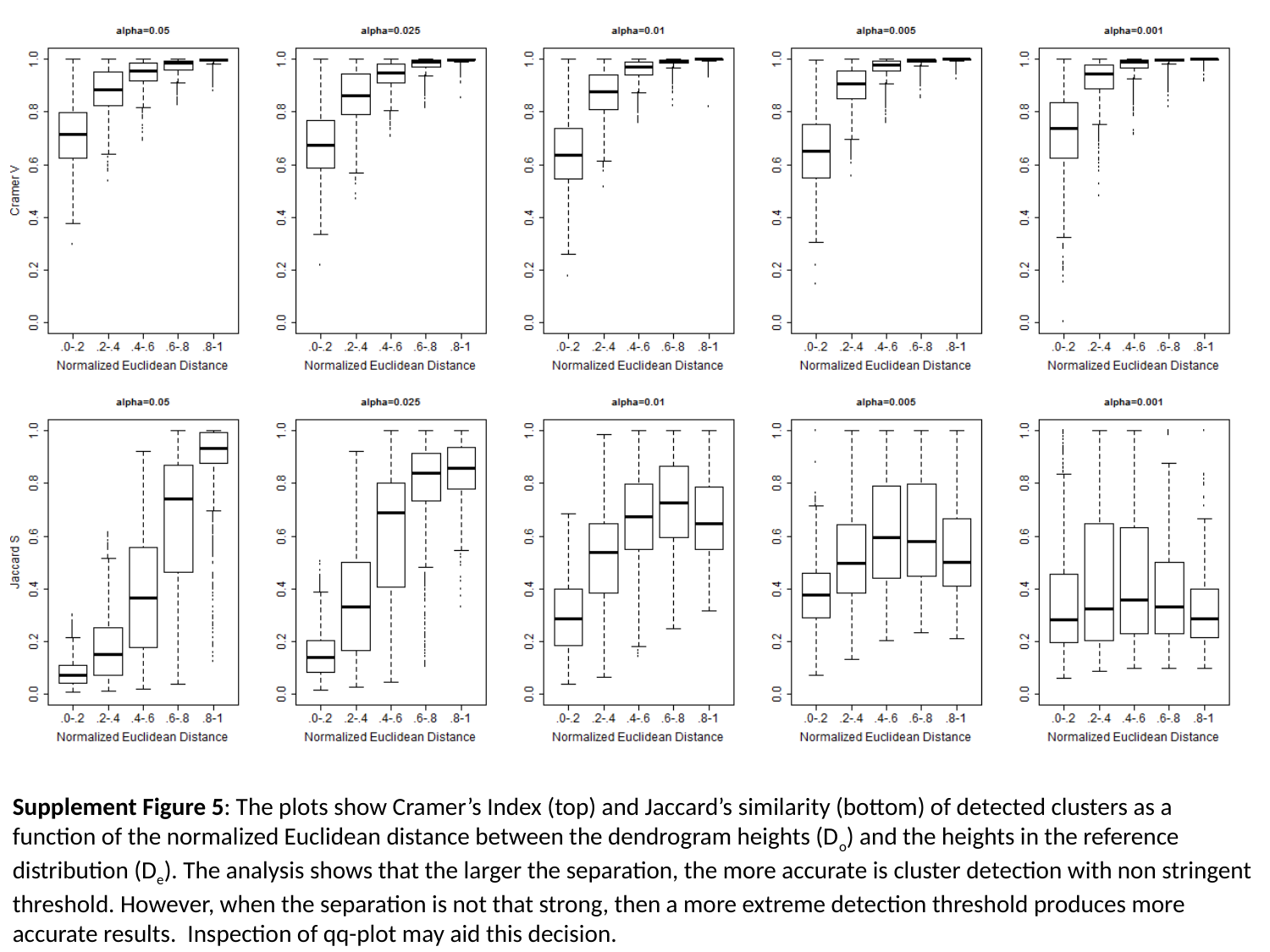

Supplement Figure 5: The plots show Cramer’s Index (top) and Jaccard’s similarity (bottom) of detected clusters as a function of the normalized Euclidean distance between the dendrogram heights (Do) and the heights in the reference distribution (De). The analysis shows that the larger the separation, the more accurate is cluster detection with non stringent threshold. However, when the separation is not that strong, then a more extreme detection threshold produces more accurate results. Inspection of qq-plot may aid this decision.

## Slide 11
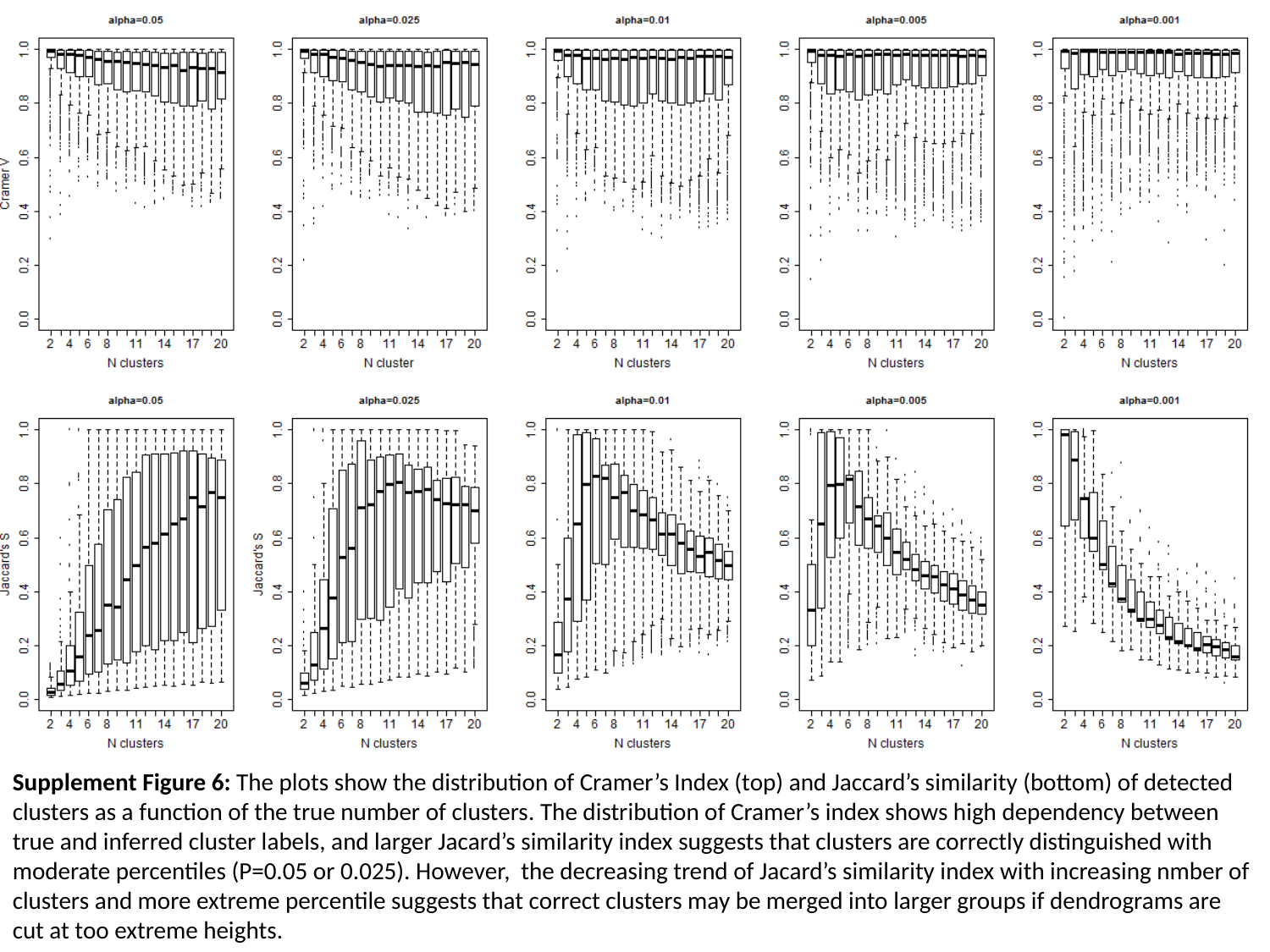

Supplement Figure 6: The plots show the distribution of Cramer’s Index (top) and Jaccard’s similarity (bottom) of detected clusters as a function of the true number of clusters. The distribution of Cramer’s index shows high dependency between true and inferred cluster labels, and larger Jacard’s similarity index suggests that clusters are correctly distinguished with moderate percentiles (P=0.05 or 0.025). However, the decreasing trend of Jacard’s similarity index with increasing nmber of clusters and more extreme percentile suggests that correct clusters may be merged into larger groups if dendrograms are cut at too extreme heights.

## Slide 12
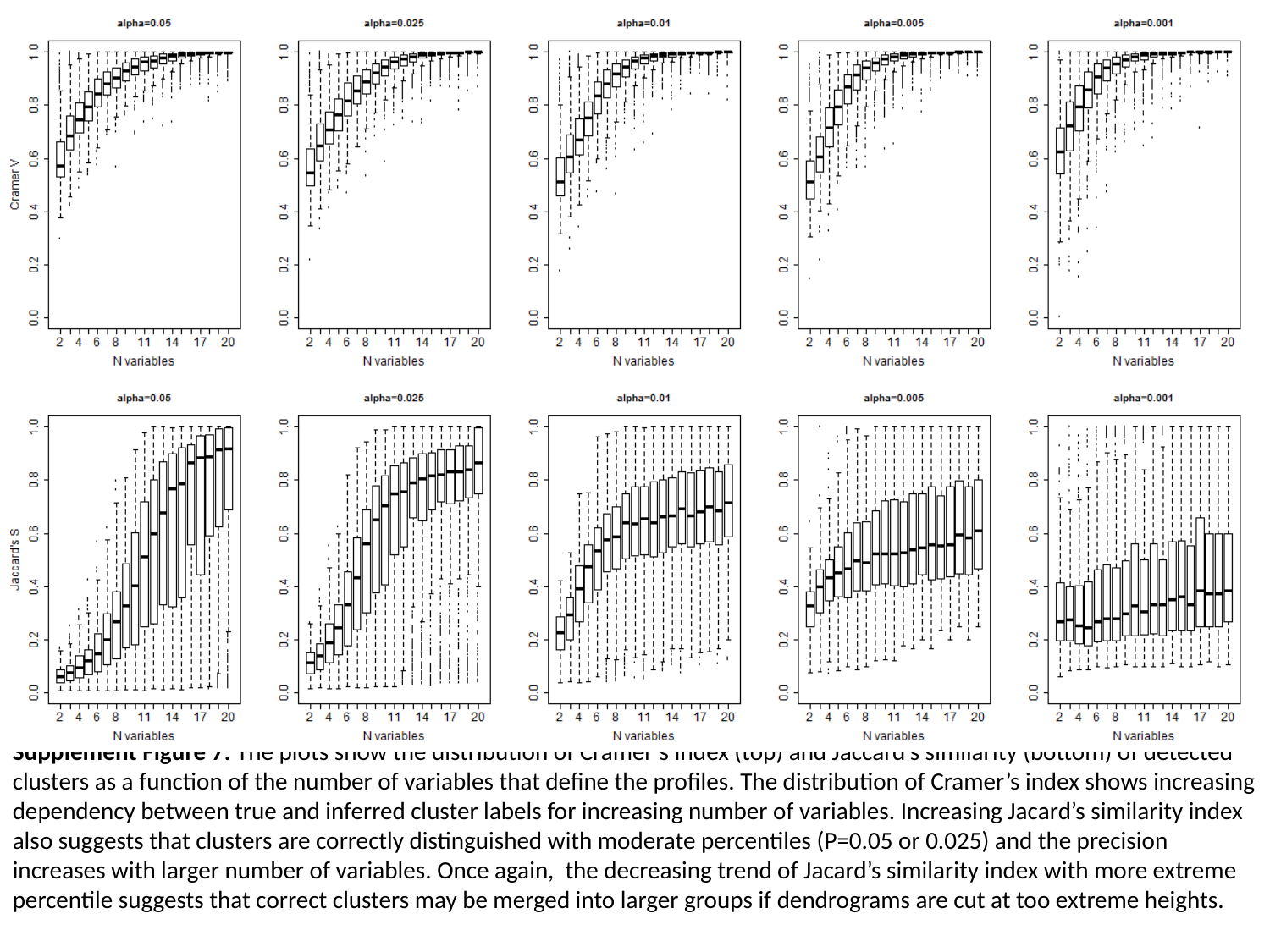

Supplement Figure 7: The plots show the distribution of Cramer’s Index (top) and Jaccard’s similarity (bottom) of detected clusters as a function of the number of variables that define the profiles. The distribution of Cramer’s index shows increasing dependency between true and inferred cluster labels for increasing number of variables. Increasing Jacard’s similarity index also suggests that clusters are correctly distinguished with moderate percentiles (P=0.05 or 0.025) and the precision increases with larger number of variables. Once again, the decreasing trend of Jacard’s similarity index with more extreme percentile suggests that correct clusters may be merged into larger groups if dendrograms are cut at too extreme heights.

## Slide 13
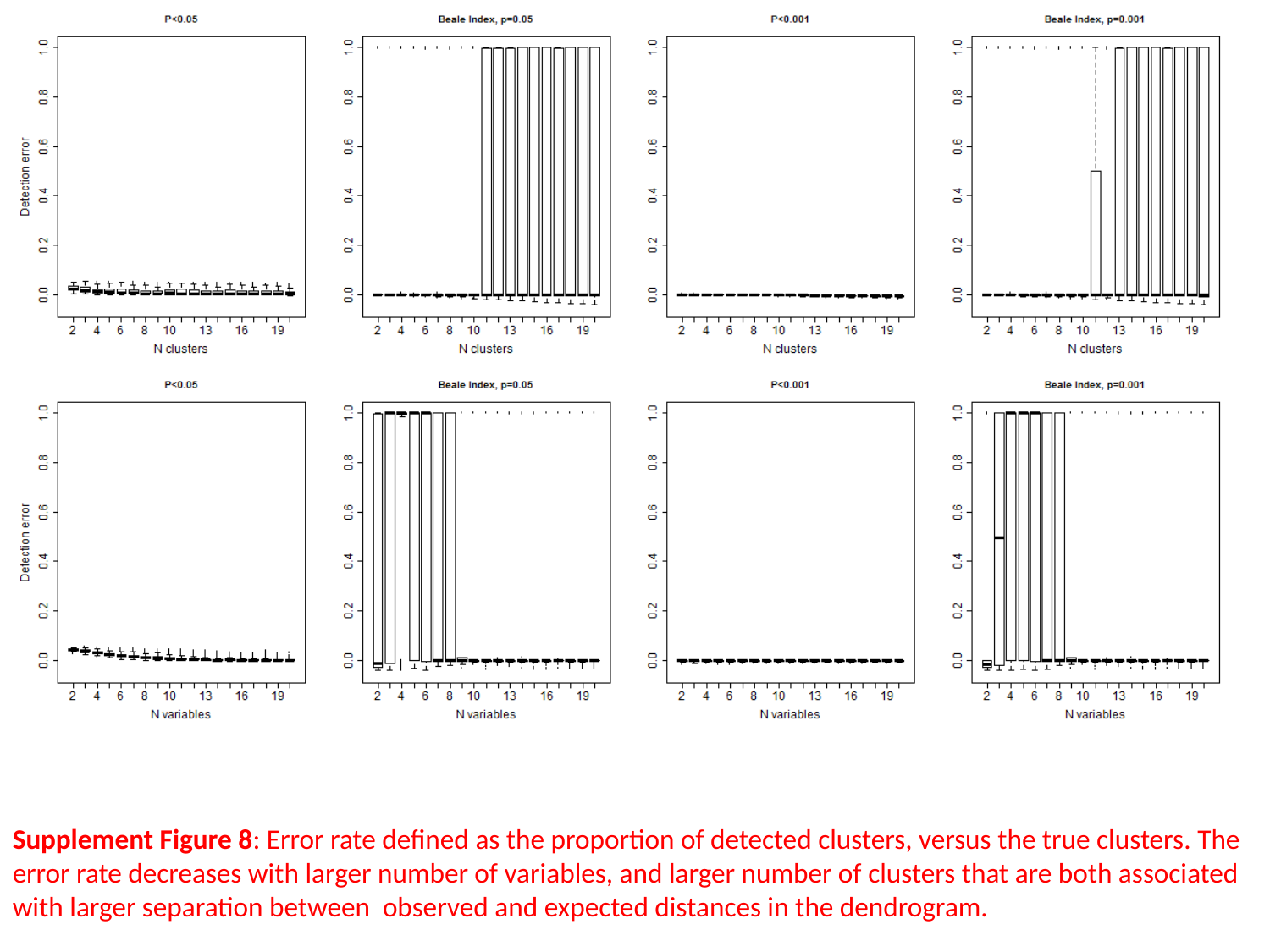

Supplement Figure 8: Error rate defined as the proportion of detected clusters, versus the true clusters. The error rate decreases with larger number of variables, and larger number of clusters that are both associated with larger separation between observed and expected distances in the dendrogram.
